# Functional Characterization of Ovine Dorsal Root Ganglion Neurons Reveal Peripheral Sensitization after Osteochondral Defect

**DOI:** 10.1523/ENEURO.0237-21.2021

**Published:** 2021-10-05

**Authors:** Sampurna Chakrabarti, Minji Ai, Katherine Wong, Karin Newell, Frances M. D. Henson, Ewan St. John Smith

**Affiliations:** 1 Department of Neuroscience, Max-Delbrück-Centrum für Molekulare Medizin (MDC), Berlin, Germany, 13125; 2 Department of Pharmacology, University of Cambridge, Cambridge, United Kingdom, CB2 1PD; 3 Department of Veterinary Medicine, University of Cambridge, Cambridge, United Kingdom, CB3 0ES

**Keywords:** knee, neuron, osteochondral defect, pain, sheep model

## Abstract

Knee joint trauma can cause an osteochondral defect (OD), a risk factor for osteoarthritis (OA) and cause of debilitating pain in patients. Rodent OD models are less translatable because of their smaller joint size and open growth plate. This study proposes sheep as a translationally relevant model to understand the neuronal basis of OD pain. A unilateral 6-mm deep OD was induced in adult female sheep. Two to six weeks after operation, lumbar dorsal root ganglia (DRG) neurons were collected from the contralateral (Ctrl) and OD side of operated sheep. Functional assessment of neuronal excitability and activity of the pain-related ion channels transient receptor potential vanilloid receptor 1 (TRPV1) and P2X3 was conducted using electrophysiology and Ca^2+^ imaging. Immunohistochemistry was used to verify expression of pain-related proteins. We observed that an increased proportion of OD DRG neurons (sheep, *N* = 3; Ctrl neurons, *n* = 15, OD neurons, *n* = 16) showed spontaneous electrical excitability (Ctrl: 20.33 ± 4.5%; OD: 50 ± 10%; *p* = 0.009, unpaired *t* test) and an increased proportion fired a greater number of spikes above baseline in response to application of a TRPV1 agonist (capsaicin) application (Ctrl: 40%; OD: 75%; *p* = 0.04, χ^2^ test). Capsaicin also produced Ca^2+^ influx in an increased proportion of isolated OD DRG neurons (Ctrl: 25%; OD: 44%; *p* = 0.001, χ^2^ test). Neither protein expression, nor functionality of the P2X3 ion channel were altered in OD neurons. Overall, we provide evidence of increased excitability of DRG neurons (an important neural correlate of pain) and TRPV1 function in an OD sheep model. Our data show that functional assessment of sheep DRG neurons can provide important insights into the neural basis of OD pain and thus potentially prevent its progression into arthritic pain.

## Significance Statement

Pain is the primary symptom of osteoarthritis (OA) and often the main reason for patients seeking medical care. Understanding pain mechanisms in OA can boost the development of disease specific pain relief. While small animals such as mouse and rat have been widely used in OA pain studies, the genetic and anatomic differences between rodents and humans can hinder clinical translation. Here, we studied pain in an early osteochondral defect (OD) model in sheep, a commonly used large animal model in OA research. We found increased excitability and transient receptor potential vanilloid receptor 1 (TRPV1) function in dorsal root ganglia (DRG) neurons innervating the site of OD. This study thus demonstrates the utility of using a large animal, such as sheep, for studying mechanisms of joint pain.

## Introduction

Osteochondral lesions are detected in ∼60% of patients who undergo knee arthroscopies (Katagiri et al., 2017). Clinically, osteochondral defects (ODs) constitute damage to bones (osteo) and cartilage (chondral) and commonly present as pain and swelling of joints after an acute injury, initial radiographs often being negative for lesions (van Dijk et al., 2010). OD is diagnosed only if pain on weight bearing persists for more than four to six weeks after injury and can also reduce the quality of life of patients to a similar extent to individuals with late-stage osteoarthritis (OA; van Dijk et al., 2010; Katagiri et al., 2017). Indeed, OD in adults is a risk factor for progression to OA, highlighting the importance of OD research to identify potential points of intervention to prevent progression to OA (Lepage et al., 2019). In OD, pain is suspected to arise from hyperexcitability of sensory nerves innervating the subchondral bone which is further amplified by secretion of inflammatory mediators from the aneural articular cartilage and synovial membrane (Lepage et al., 2019). However, direct evidence of sensory nerve hyperexcitability in OD and the mechanisms involved in such peripheral sensitization is lacking.

Electrophysiological recording from isolated dorsal root ganglia (DRG) neurons (location of the cell bodies of sensory nerves innervating the joint) harvested from rodents is commonly used to study peripheral sensitization. However, rodent models of surgical OD are difficult to create and less translatable because of their open growth plate and smaller cartilage volume (rats: 2.17 mm^3^) compared with humans (552 mm^3^; Meng et al., 2020). In contrast, large animals, such as sheep, share a similar cartilage volume (359 mm^3^) and knee joint anatomy to humans leading to successful OD induction in joints as evidenced by histologic scoring and reduced activity (Newell et al., 2018; Meng et al., 2020). Therefore, surgically inducible OD in sheep provides a suitable model for orthopedic research, although this approach has been criticized because of large inter-animal variability of cartilage thickness (Ahern et al., 2009).

Although pain is a major symptom, most OD research has focused on developing strategies for bone and cartilage regeneration, such as implantation of biomaterials in large animal models (Meng et al., 2020). Whether such regeneration strategies also decrease any neuronal sensitization that occurs in OD, and hence pain, is largely unknown because of the lack of expertise in isolation and recording of DRG neurons from large animals. In this study, we provide evidence of peripheral sensitization in an OD model of the sheep stifle joint by electrophysiological recording and Ca^2+^ imaging of isolated DRG neurons. Such an in vitro experimental paradigm could be used to identify translatable pain targets for OD and OA and as an outcome measure of future pain therapeutics and cartilage regeneration technologies.

## Materials and Methods

This study was approved by the [Author University] Animal Welfare and Ethical Review Body and the United Kingdom Home Office (Project License 70/8165).

### Animals

Six skeletally mature female Welsh Mountain sheep (3.2 ± 0.8 years, 40–44 kg) housed in flocks under natural conditions with the same feed, husbandry and location were included in the study. All animals were housed in flocks outside under natural conditions with the same feed, husbandry, and location.

### Animal anesthesia, preparation, and surgical technique

Sheep were anaesthetized by intravenous injection of thiopentone (3 mg/kg) and maintained using inhalation anesthesia (a mixture of isoflurane, nitrous oxide, and oxygen). Perioperative analgesia was provided by intramuscular injection of carprofen (4 mg/kg). Antibiotic prophylaxis (procaine penicillin) was given through intramuscular injection. All animals used went through the identical surgical procedure under strict aseptic conditions. Each stifle joint was physically examined for abnormality under anesthesia and animals with gross joint instability or pathology were excluded from the study.

The OD was created on the left stifle joint of experimental sheep. Each animal was placed in a dorsal recumbency position following surgical preparation and the left stifle joint was opened through a parapatellar approach. The patellar fat pad was then elevated to access the medial femoral condyle (MFC) where a 6-mm deep, 8-mm wide OD was created using a hand drill. Following surgery, operated animals were given carprofen (4 mg/kg) for postoperative pain measurements for 3 d and were kept in small pens for 48 h to reduce ambulation before allowing them to fully bear weight. Sheep were then housed in large pens or outdoor fields with normal ambulation before being killed by intravenous injection of 40-ml 20% (w/v) pentobarbitone sodium two to six weeks after surgery, since postoperative pain is likely to have subsided by two weeks and very little cartilage healing takes place by six weeks (Xu and Brennan, 2010; Lydon et al., 2019). Two cohorts, each of three sheep, were used in this study for immunohistochemistry and electrophysiology experiments, respectively. All three sheep in each cohort were operated at the same time to reduce variability and killed after two to six weeks. The time window of two to six weeks was chosen because of resources/personnel making it impossible to process tissue from all three sheep simultaneously, as well as based on the evidence that minimal cartilage healing takes place within six weeks. Macroscopic scoring of the joint was caried out blindly according to the International Cartilage Repair Society guidelines (van den Borne et al., 2007).

### DRG neuron isolation and culture

DRG in the lumbar region (L3–L4) were dissected from operated (OD) and non-operated (Ctrl) side of three sheep immediately following their killing and placed in ice cold dissociation media [L-15 Medium (1×) + GlutaMAX-l (Life Technologies) with 24 mm NaHCO_3_ supplement] for transport. DRG dissection was done in line with previously reported procedure on sheep and other species (Malin et al., 2007; Russo et al., 2010; Fadda et al., 2016) Briefly, sheep were placed on the operating table in the posterior position and their midline fur was shaved using a veterinary clipper. A surgical scalpel was then used to cut open the skin, retract the obliques and latissimus muscles, after which a bone saw was used to remove the lumbar (L2–L5) part of the vertebrae en bloc. A dorsal laminectomy was then performed using bone saw to expose the spinal cord and DRG. Exposed DRG were carefully lifted with a forceps while dissecting scissors were used to simultaneously cut the spinal root and peripheral nerve rami to free the DRG.

The collected DRG were cut into ∼3 mm^3^ pieces and submerged in 3 ml collagenase solution [1 mg/ml Type I collagenase A (Sigma) with 6 mg/ml bovine serum albumin (BSA; Sigma)] and placed on a shaker (30 rev/min) for 10 min before 15-min incubation in a 37°C incubator. Collagenase solution was then replaced with 3 ml prewarmed trypsin solution [1 mg/ml trypsin (Sigma) with 6 mg/ml BSA in dissociation media] and placed on a shaker for 5 min before 30-min incubation at 37°C. After removal of the trypsin solution, prewarmed culture media [dissociation medium with 10% (v/v) fetal bovine serum, 2% penicillin/streptomycin and 38 mm glucose] was added to the DRG and the solution transferred to a 15 ml falcon tube for gentle mechanical trituration with a 1 ml Gilson pipette followed by brief centrifugation (160 × *g*, 30 s; Biofuge primo, Heraeus Instruments). Supernatant containing dissociated DRG neurons was collected in a fresh tube. This dissociation step was repeated for five times until 10 ml of supernatant was collected. Collected supernatant was centrifuged at 160 × *g* for 5 min for neuron pelleting, which was then resuspended in 250 μl DRG culture media and plated on poly-D-lysine and laminin coated glass bottomed dishes (MatTek, P35GC-1.5-14-C) for 3 h to allow neuron to attach. An additional 2 ml of culture medium was added to each culture dish following neuron attachment. All DRG neurons were placed in incubator (37°C, 5% CO_2_) overnight (8–10 h) before electrophysiology and Ca^2+^ imaging recordings.

### Whole-cell patch clamp electrophysiology

DRG neuron recordings were made following overnight incubation. Tested neurons were bathed in extracellular solution (ECS; 140 mm NaCl, 4 mm KCl, 2 mm CaCl_2_, 1 mm MgCl_2_, 4 mm glucose, and 10 mm HEPES, adjusted to pH 7.4 with NaOH) and recorded from using an EPC-10 amplifier (HEKA) and Patchmaster software (HEKA). Glass pipettes were pulled (P-97, Sutter Instruments) from borosilicate glass capillaries with a resistance of 3–6 MΩ. A ground electrode was placed in the neuron bath to form a closed electric circuit with patching pipette loaded with intracellular solution: 110 mm KCl, 10 mm NaCl, 1 mm MgCl_2_, 1 mm EGTA, 10 mm HEPES, 2 mm Na_2_ATP, and 0.5 mm Na_2_GTP, adjusted to pH 7.3 with KOH. Neurons were held at −60 mV with pipette and membrane resistance compensated. Resting membrane potential (RMP), cell resistance and capacitance were recorded on current-clamp mode prior than any testing protocols. Action potentials (APs) were recorded in current-clamp mode either without current injection (to record spontaneous firing) or following stepwise current injection (to evoke firing), current being injected from 100 to 1000 pA for 80 ms through 20 steps and the first evoked AP was analyzed. AP threshold, half peak duration (HPD; ms), amplitude, afterhyperpolarization (AHP) duration (ms), and AHP amplitude (mV), were measured using FitMaster (HEKA) software or IgorPro software (Wavemetrics) as previously described (Chakrabarti et al., 2018).

Transient receptor potential vanilloid receptor 1 (TRPV1) and purinergic (P2X3) ion channel agonists [capsaicin (1 μm) and αβ m-ATP (30 μm); Sigma] were applied to DRG neurons for 10 s to determine their ability to evoke AP generation in current clamp mode. Both agonist solutions were made up in pH 7.4 ECS from respective stock solution (1 mm capsaicin stock in 100% ethanol, Sigma-Aldrich; and 5 mm αβ m-ATP stock in 100% ethanol). The average Δ spike in response to each agonist was calculated by spike numbers (normalized by subtracting spike numbers at pH 7.4) divided by agonist application time.

**Figure 1.**
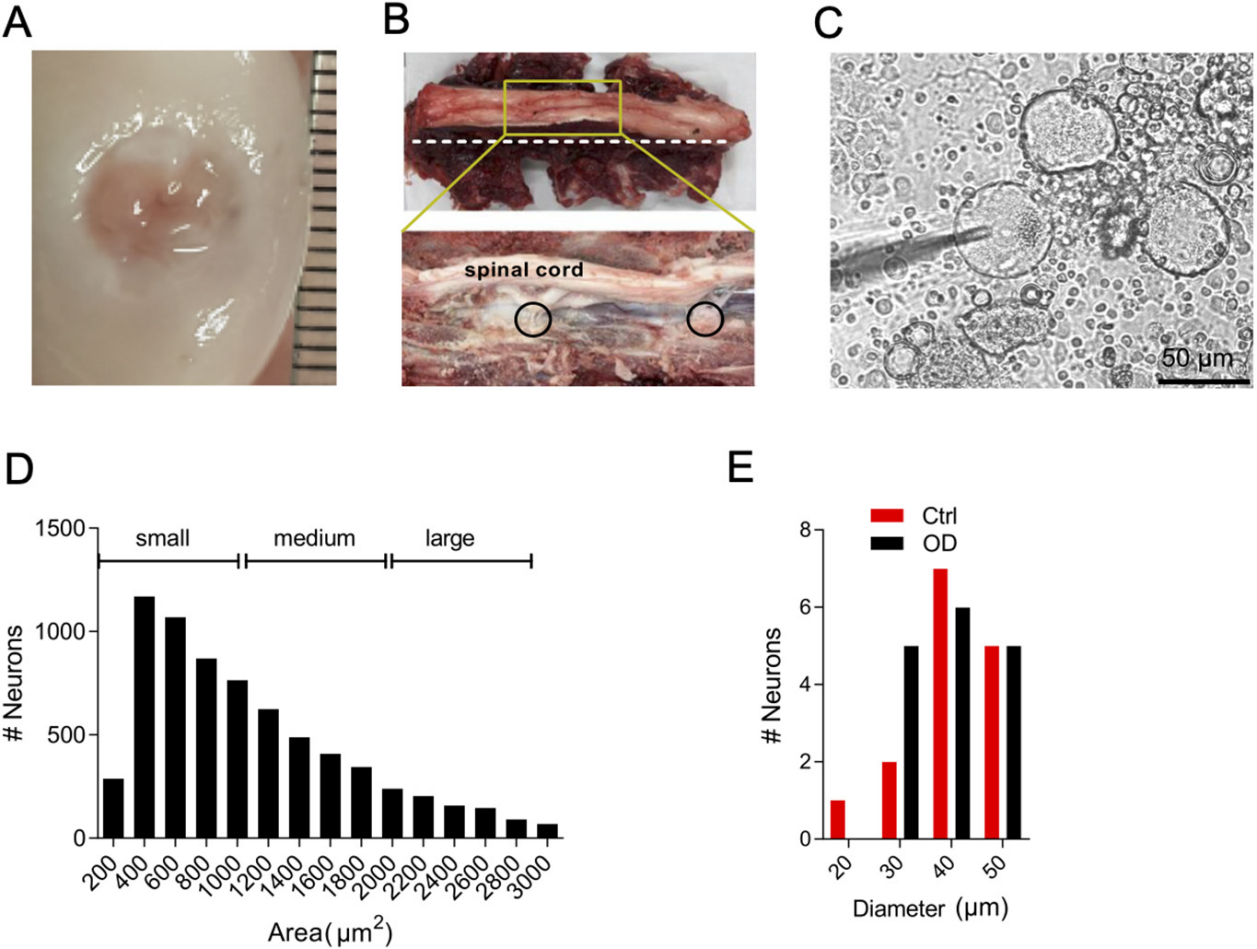
Characterizations of defect and sheep DRG neurons. ***A***, Photograph of OD created in sheep joint. ***B***, Photograph showing intact (top) lumbar region of a sheep spine and after transverse section (white dotted dissection line, bottom) to expose DRG (black circles). ***C***, Acutely dissociated sheep DRG neurons in culture. ***D***, Histogram showing area of each sheep DRG neuron imaged from whole DRG section, and the criteria used in this article for assigning neurons into small, medium, and large category. ***E***, Histogram of neuronal diameters on which whole-cell patch clamp was performed.

Current-voltage relationships were obtained using a standard voltage-step protocol under voltage-clamp mode (Fig. 1*G*; da Silva Serra et al., 2016). Cells were held at −120 mV for 240 ms before stepping to the test potential (−50 to +40 mV in 10-mV increments) for 40 ms (Fig. 1*H*). Voltage was returned to holding potential (−60 mV) for 200 ms between sweeps. Leak subtraction was applied to minimize capacitive currents. Step current density was calculated by minimum (inward) and maximum (outward) current amplitude [pA; normalized by subtracting average baseline amplitude (100 ms) at –120 mV] dividing cell capacitance (pF). Calculated current density (pA/pF) was then plotted against corresponding step voltage (mV) as voltage-current relation and fitted in Igor Pro using a single or double Boltzmann equation.

### Ca^2+^ imaging

DRG neurons were incubated in 10 μm of the Ca^2+^ indicator Fluo-4 A.M. (diluted from a 10 mm stock solution in DMSO in ECS; Invitrogen) for 30 min at room temperature (21°C). Neurons were then washed with ECS and placed on microscope (Nikon Eclipse Tie–S, Nikon) for imaging. Fluro-4 fluorescence was excited by a 470 nm LED (Cairn Research) and images were captured by a digital camera (Zyla cSMOS, Andor) at 1 Hz with 50-ms exposure time using Micro-Manager software (v1.4; NIH).

The same ion channel agonist solutions used in electrophysiology and 50 mm KCl (to serve as a positive control) were applied to neurons following an established perfusion protocol: 10 s ECS wash following 10-s agonist application and another 90 s wash in ECS. All solutions were perfused through a gravity-driven 12-barrel perfusion system. A 3 min interval was applied to allow the neurons to return to their resting state among each perfusion.

Data analysis was conducted following an in house protocol (Chakrabarti et al., 2020b). Briefly, KCl positive cells and one black background were drawn manually as a region of interest (ROI) using ImageJ software and the mean gray value of selected ROIs in sequence was extracted. Extracted data were then analyzed by lab-developed R toolbox (https://github.com/amapruns/Calcium-Imaging-Analysis-with-R) to calculate the change in Ca^2+^ influx (normalized to peak KCl response (ΔF/Fmax) with background subtraction) and the percentage of agonist respondent cells (cells with ΔF/Fmax value <0.001 and peak after 30 s were deleted manually).

### Immunohistochemistry

In a separate cohort of *N* = 3 sheep, L3–L4 DRG from contralateral and ipsilateral sides of OD animals were collected as described above. Collected DRG were immediately fixed in Zamboni’s fixative (4% paraformaldehyde and picric acid) for 1 h and transferred to a 30% (w/v) sucrose solution for overnight incubation at 4°C. Processed DRG were then embedded in Shandon M-1 embedding matrix (Thermo Fisher Scientific), snap frozen in liquid nitrogen and stored at −80°C. Embedded DRG were sectioned by a Leica Cryostat (CM3000), mounted on Superfrost Plus microscope slides (Thermo Fisher Scientific) and stored at −20°C until staining. One to three sections were chosen randomly from both operated and non-operated sides for analysis. Staining was conducted by following an established protocol (Chakrabarti et al., 2018) and performed blindly by K.W. Anti-CGRP (1:5000, Sigma C8189, anti-rabbit polyclonal) and anti-P2X3 (1:1000, Alomone APR016, anti-rabbit polyclonal) primary antibodies were used in combination with an Alexa Fluor 488 anti-rabbit conjugated secondary antibody (1:1000, Invitrogen A21206) and Alexa Fluor 568 anti-mouse conjugated secondary antibody (1:500, Invitrogen A-11031). Using secondary antibodies alone resulted in no staining. The mean gray value of each DRG neuron was measured in ImageJ and a custom-made R toolkit (https://github.com/amapruns/Immunohistochemistry_Analysis) was used to identify positive neurons with manual validation as previously described (Chakrabarti et al., 2020b). In brief, a normalized distribution of neurons with the least mean gray value from each section was computed (distribution of minima). All neurons that had a mean gray value >2 SDs from the average of the distribution of minima were scored positive.

### Statistics

All figures presented were analyzed and graphed in GraphPad Prism 8 or IgorPro software unless stated otherwise. Data shown as mean ± SD. Two group comparisons were carried by Student’s unpaired *t* test, and percentage comparison was done by χ^2^ test; *p* < 0.05 was considered significant. *N* = 3 sheep (two to six weeks post-OD) were used for electrophysiology and Ca^2+^ imaging while another cohort of *N* = 3 sheep were used for immunohistochemistry.

## Results

A 6 mm OD was created unilaterally on the femoral condyle of sheep (*N* = 6) stifle joint which resulted in cartilage damage (Fig. 1*A*). The damage was assessed macroscopically (Table 1). Sheep lameness was scored qualitatively twice daily using a clinical grading system in the first week of surgery and thereafter if lameness was observed during daily inspections. At the time of killing, all sheep scored grade 0 (no lameness). Pain is the major symptom of OD in humans, and it was previously shown that sheep reduce their free movement (measured by activity trackers) after OD, presumably because of pain (Newell et al., 2018). It has also been reported that synovial fluid from patients with painful OA can increase the excitability of mouse DRG neurons (Chakrabarti et al., 2020b). Consequently, we hypothesized that the isolated DRG neurons from the OD side of sheep would show hyperexcitability. To test the hypothesis, we harvested, cultured and performed patch-clamp recording on DRG neurons isolated from the Ctrl (*n* = 15) and OD (*n* = 16) sides of three operated sheep (Fig. 1*B*). We recorded from small-medium sized putative nociceptors (<1000 μm^2^ in area; Fig. 1*C–E*).

**Table 1:** Scores of ODs in sheep by International Cartilage Repair Society (ICRS) macroscopic scoring system

Sheep #	ICRS macroscopic score (appearance of joint postmortem)
1	3
2	2
3	2
4	2
5	3
6	2

### OD induces hyperexcitability in sheep DRG neurons

**Figure 2.**
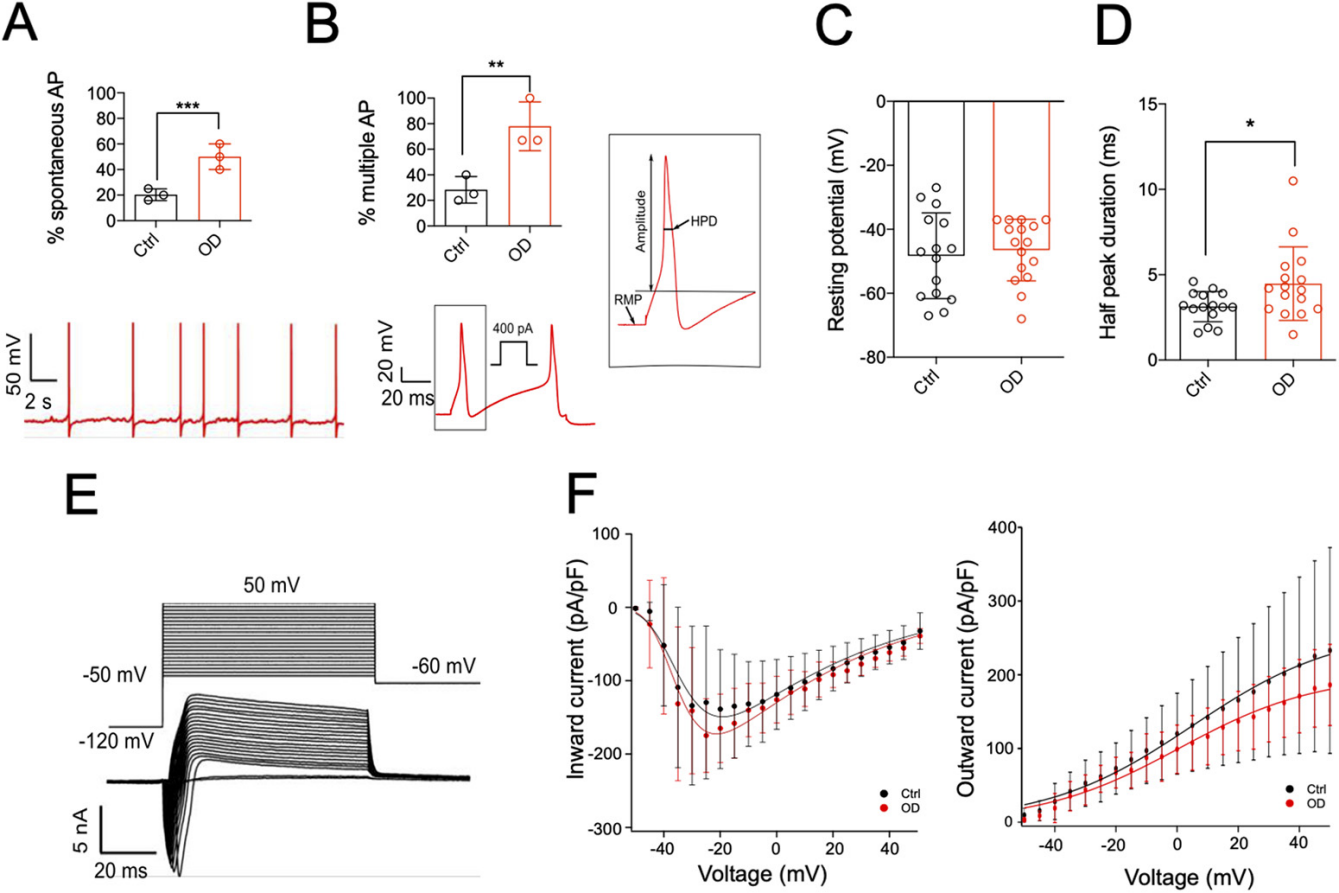
OD neurons are more excitable than ctrl neurons. ***A***, Percentage of neurons showing spontaneous activity in ctrl and OD condition (top) and a representative OD neuron with spontaneous activity (bottom). ***B***, Percentage of neurons firing multiple APs on current injection (top) and a representative trace of multiple firing in response to 400 pA injected current (bottom). ***C***, Schematic representation of AP properties (left) and distribution of HPD (right) and (***D***) RMP in ctrl and defect conditions. ***E***, Representative traces of currents evoked from a neuron at different voltages and (***F***) plots of the inward and outwards currents in ctrl (black) and defect (red) conditions; *p < 0.05, **p < 0.01, ***p < 0.001, unpaired *t* test. See Extended Data Figure 2-1 for breakdown of data according to weeks post-OD.

We observed that neurons from the OD side exhibited enhanced spontaneous AP firing in the absence current injection (Ctrl: 20.33 ± 4.5%; OD: 50 ± 10%; *p* = 0.009, unpaired *t* test; Fig. 2*A*) and greater likelihood of multiple AP firing after current injection (Ctrl: 28.33 ± 10.41%; OD: 78 ± 19.05%; *p* = 0.01, unpaired *t* test; Fig. 2*B*). These results suggest that OD enhances nociceptor excitability. Notably, the AP threshold in Ctrl and OD neurons was ∼100 pA (Table 2), which is lower than that reported for murine DRG neurons (Chakrabarti et al., 2018). Additionally, AP HPD was increased in neurons isolated from the OD side (Ctrl: 3.13 ± 0.88%; OD: 4.48 ± 2.15%; *p* = 0.03, unpaired *t* test; Fig. 2*D*), while no change in RMP (Ctrl: −48.27 ± 13.39%; OD: −46.5 ± 9.63%; *p* = 0.67, unpaired *t* test; Fig. 2*C*) or other AP properties was observed (Table 2). Increase in HPD is suggestive of increased voltage-gated Ca^2+^ (Ca_v_) and Na_v_1.8 channel function (Blair and Bean, 2002), but no difference in macroscopic inward (mediated by Na_v_ and Ca_v_) or outward (mediated by K_v_) current–voltage relationships was found between Ctrl and OD conditions (Fig. 2*E*,*F*); recordings of isolated Na_V_, Ca_V_, and K_V_ currents were not conducted. Taken together, our data suggest that up to six weeks after OD, sheep DRG neurons show increased excitability, which is a correlate of pain, and this increase in excitability was not because of overt changes in summed, macroscopic inward or outward currents generated by voltage-gated ion channels.

**Table 2:** AP properties of sheep DRG neurons in Ctrl and OD groups

	Ctrl (*n* = 15)		OD (*n* = 16)	
	Mean	SD	Mean	SD
Diameter (μm)	39.5	8.309	39.5	7.611
Capacitance (pF)	71.0	41.24	80.5	47.24
RMP (mV)	−48.3	13.39	−46.5	9.633
Threshold (pA)	116.7	155.5	100.0	225.8
HPD (ms)	3.1*	0.8886	4.5	2.151
AHP amplitude (mV)	14.0	7.548	14.0	9.126
Amplitude (mV)	101.6	18.17	103.5	11.17

*p < 0.05, unpaired *t* test.

10.1523/ENEURO.0237-21.2021.f2-1 Extended Data Figure 2-1 Extended data supporting Figure 2. Data points from main figures separated according to weeks post-OD. ***A***, AP properties of sheep DRG neurons. Percentage of sheep DRG neurons which evoked AP upon application of capsaicin (***B***) and αβ m-ATP (***C***). ***D***, Percentage and magnitude of Ca^2+^ influx of sheep DRG neurons in response to capsaicin and αβ m-ATP. ***E***, percentage of P2X3 and CGRP positive sheep DRG neurons as assessed using immunohistochemistry. In all the panels, black = control, red = OD, circle = data from sheep two weeks post-OD, square = data from sheep four weeks post-OD and, triangle = data from sheep six weeks post-OD; *p < 0.05, **p < 0.01, unpaired *t* test. Download Figure 2-1, TIF file.

### Sheep DRG neurons show increased TRPV1 function after OD

**Figure 3.**
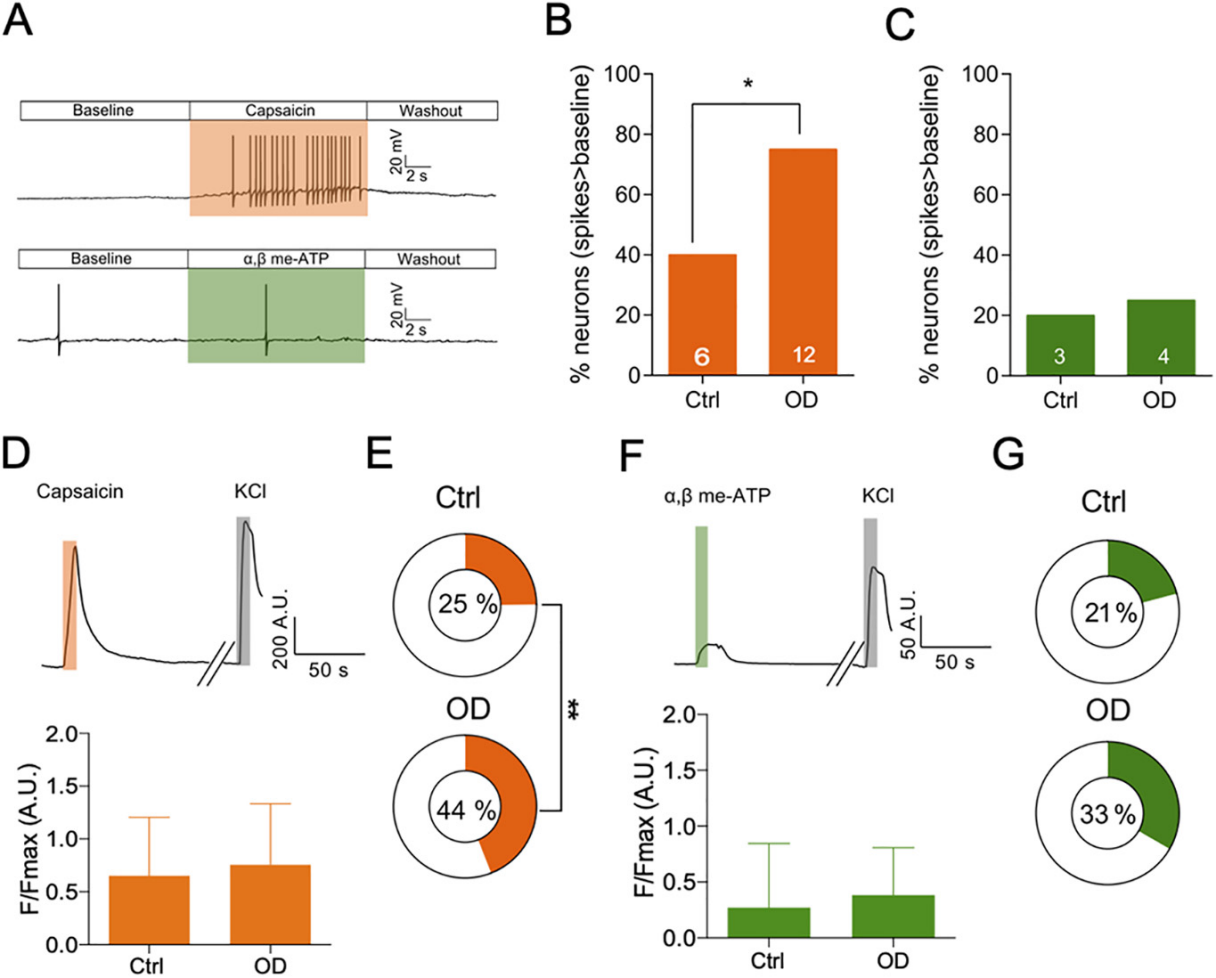
OD neurons have increased TRPV1 function than ctrl neurons. ***A***, Patch-clamp traces showing a representative DRG neuron from the ctrl side firing AP spikes above baseline and same as baseline in response to capsaicin and αβ me-ATP, respectively. ***B***, Bar graph showing percentage of neurons with above baseline firing activity in response to capsaicin ***C***, Bar graph showing percentage of neurons with above baseline firing activity in response to αβ me-ATP. Numbers on the bars represent number of neurons in each condition firing above baseline. ***D***, top, Representative Ca^2+^ trace from a neuron responding to capsaicin and KCl (positive control). Bottom, Magnitude of Ca^2+^ influx in response to capsaicin (Ctrl, *n* = 35, defect, *n* = 48). ***E***, Percentage of neurons responding to capsaicin in each condition. ***F***, top, Representative Ca^2+^ trace from a neuron responding to αβ me-ATP and KCl (positive control). Bottom, Magnitude of Ca^2+^ influx in response to αβ me-ATP (ctrl, *n* = 24, defect, *n* = 23). ***G***, Percentage of neurons responding to αβ me-ATP in each condition. *p < 0.05, unpaired t-test.

In addition to voltage-gated ion channels, increased functionality of algogen-sensing ion channels can also cause nociceptor hyperexcitability (Chakrabarti et al., 2018). We tested chemical agonists of TRPV1 and P2X3 ion channels (capsaicin and αβ me-ATP, respectively) and observed that sheep DRG neurons also respond to these known algogens by firing AP (Fig. 3*A*) similar to neurons isolated from mouse and human DRG (Davidson et al., 2014). Additionally, we observed that the proportion of OD neurons firing above baseline on application of capsaicin was significantly higher than in Ctrl neurons, implicating an increase in TRPV1 function [Ctrl: 40% (6/15); OD: 75% (12/16); *p* = 0.04, χ^2^ test; Fig. 3*B*]. However, αβ me-ATP produced above baseline firing in similar proportion of neurons isolated from both Ctrl and OD sides, thus arguing against a significant role of P2X3 channel in OD pain [Ctrl: 20% (3/15); OD: 25% (4/16); *p* = 0.73, χ^2^ test; Fig. 3*C*]. As only a small population of DRG neurons can be tested through patch-clamp electrophysiology (Ctrl, *n* = 15 vs OD, *n* = 16). We next performed Ca^2+^ imaging on these neurons to investigate the tested ion channel functions in a larger neuron population (Ctrl, *n* = 141 vs OD, *n* = 109). We found an increased proportion of OD neurons respond to capsaicin [Ctrl: 25% (35/141), OD: 44% (48/109), *p* = 0.001, χ^2^ test; Fig. 3*D*,*E*], while the number of neurons responding to αβ me-ATP was not significantly different between Ctrl and OD groups [Ctrl: 21% (24/115), OD: 33% (23/69), *p* = 0.06, χ^2^ test; Fig. 3*F*,*G*].

### IHC revealed similar P2X3+ neuron populations following OD

**Figure 4.**
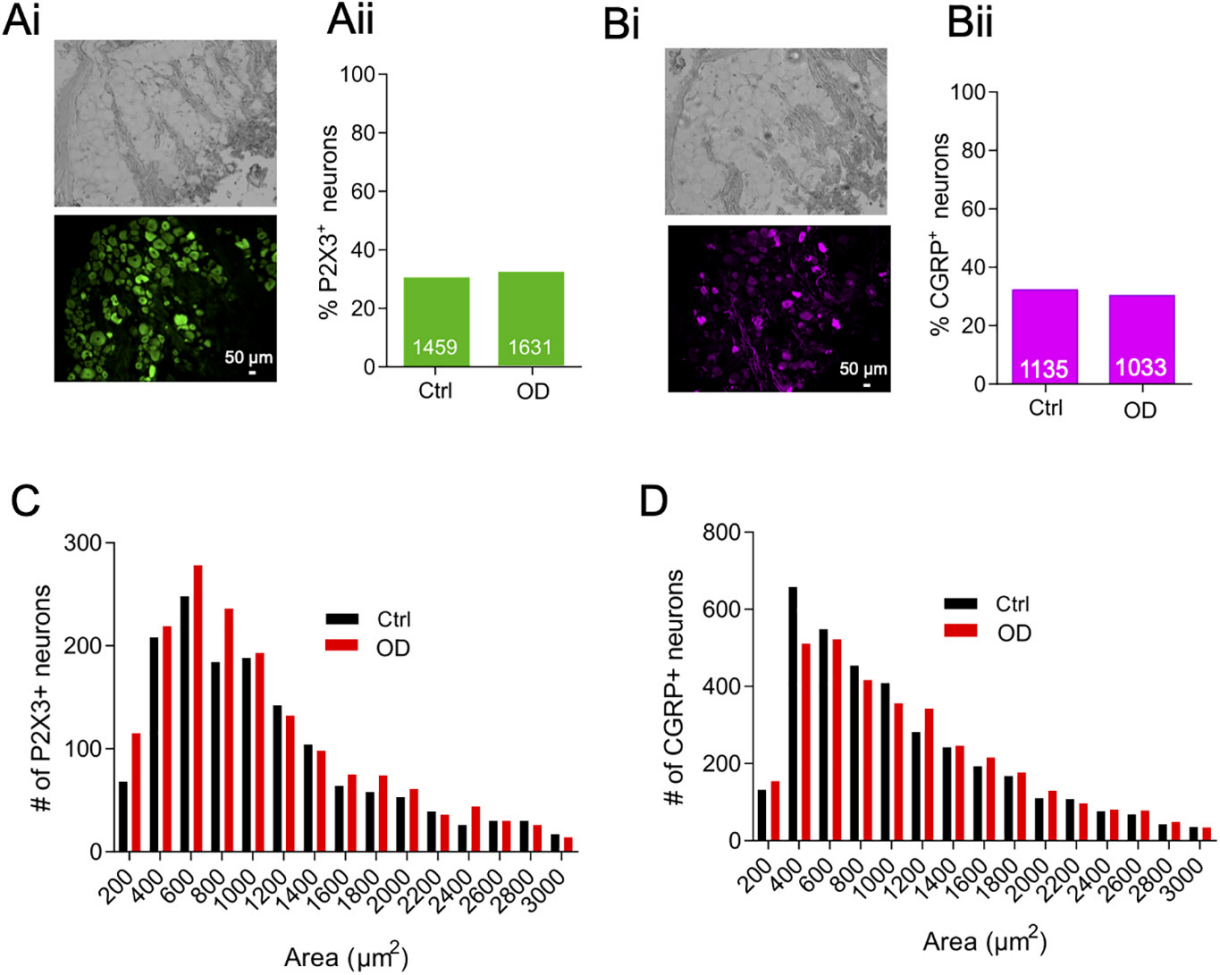
OD and ctrl DRGs showed similar proportion of P2X3 and CGRP positive neurons. ***Ai***, Representative brightfield (top) and anti-P2X3-antibody stained (bottom) image of a whole-sheep DRG in cross-section along with the percentage of neurons positive for P2X3 (***Aii***). ***Bi***, Representative brightfield (top) and anti-CGRP-antibody stained (bottom) image of a whole-sheep DRG in cross-section along with the percentage of neurons positive for CGRP (***Bii***). Histograms of cross-sectional areas of neurons stained positive by anti-CGRP (***C***) and anti-P2X3 (***D***) antibodies. Numbers on the bars represent neurons positive for the respective antibody staining; *p < 0.05, **p < 0.01, χ^2^ test.

Increased ion channel function usually correlates with increased ion channel expression. We showed using IHC on sections of whole DRG that the proportion of P2X3+ neurons was similar (∼30%) in Ctrl and OD conditions [Ctrl: 30.66% (1459/4758), OD: 31.77% (1631/5133), *p* = 0.23, χ^2^ test; Fig. 4*Ai*,*Aii*]. However, protein level expression of TRPV1 could not be validated because of the unavailability of a specific antibody that reliably detected sheep TRPV1 (tested antibodies listed in Table 3). Finally, we probed the expression of the pronociceptive neuropeptide, CGRP, in DRG sections because an increase in TRPV1 expression can in turn induce production of CGRP (Iyengar et al., 2017). However, the proportion of CGRP+ neurons was ∼30% in both OD and Ctrl conditions as observed before (Russo et al., 2010), suggesting that the proportion of peptidergic, CGRP+ neurons is unchanged in OD [Ctrl: 32.2% (1135/3524), OD: 30.26% (1033/3413), *p* = 0.09, χ^2^ test; Fig. 4*Bi*,*Bii*]. Histogram analysis of neuron size in both P2X3+ neurons (Fig. 4*C*) and CGRP+ neurons (Fig. 4*D*) revealed that majority of positive neurons were small size neurons that are likely to be nociceptors.

**Table 3:** List of TRPV1 antibodies tested on sheep DRGs

Antibody	Description	Supplier	Catalog
Anti-TRPV1, mouse monoclonal	Primary	Proteintech	66983-1-Ig
Anti-TRPV1, rabbit polyclonal	Primary	Abcam	Ab3487
Anti-Trpv1, rabbit polyclonal	Primary	Abcam	Ab31895
Anti-TRPV1, guinea pig polyclonal	Primary	Alomone	AGP-118

Taken together, our data provide support that OD causes hyperexcitability of sheep DRG neurons and that increased function of TRPV1 is part of the sensitization process. Since TRPV1 is also an important pain target in OA, TRPV1 antagonists used for pain control in OD might also help prevent pain in cases of OD that develop into OA.

## Discussion

It has been previously proposed that large animals, such as sheep, can be leveraged as translational models to investigate mechanisms of joint pain in vitro because of their larger joint anatomy and DRG neuron diameter compared with rodents (Chakrabarti et al., 2020a). We provide proof using the ovine OD model (which is more translatable than rodent OD models) that it is possible to study neuronal constructs of peripheral sensitization in large animals using tools more widely used for analyzing rodent DRG neurons. For example, we show using whole-cell patch clamp electrophysiology and Ca^2+^ imaging that sheep DRG neurons can be activated by agonists of nociceptive ion channels TRPV1 and P2X3, thus indicating their functional presence.

Importantly, we found using whole-cell patch clamp that an increased proportion of OD neurons fired spontaneous AP and multiple APs within 80 ms in response to injected current compared with Ctrl neurons. The increase in AP firing did not appear to be associated with a significant change in voltage-gated ion channel function, however, further studies are needed to determine if, for example, changes in the activity of hyperpolarization-activated cyclic nucleotide-gated (HCN) channels occur, HCNs having been implicated in sensory nerve firing and pain (Tsantoulas et al., 2017). We also observed increased AP firing on administration of the TRPV1 agonist capsaicin in OD neurons, along with an increased proportion of OD neurons responding to capsaicin using Ca^2+^ imaging. These data suggest that TRPV1-mediated depolarization can increase AP firing in sensory neurons to a greater extent after OD, which is consistent with mechanistic studies showing a TRPV1-anoctamin 1 interaction that increases prolonged glutamate release to induce pain-related behaviors (Takayama et al., 2015). Furthermore, our data are consistent with peripheral sensitization observed in models of joint pain (Chakrabarti et al., 2018), implying that similar nociceptive mechanisms are also at play before induction of arthritis. Therefore, TRPV1 antagonists have the potential to ameliorate or perhaps prevent onset of arthritic pain. At present, no such TRP antagonists are clinically approved, but mavatrep (TRPV1 antagonist), has shown promising results in clinical trials of OA (Manitpisitkul et al., 2018). Since our DRG samples were collected two to six weeks after OD, a limitation of the present study is the potential changes in nociceptive molecular signature between two and six weeks after OD. Although we cannot rule-out such changes based on our current experimental paradigm, a detailed analysis of data points derived from two, four, and six weeks after OD (Extended Data Fig. 2-1), shows no apparent time-related change in nociceptor activity two to six weeks post-OD. Furthermore, although postoperative pain normally resolves within a week (Ahern et al., 2009), a limitation of the present study is lack of a sham control group (not permitted by our animal ethics) and therefore, some effects observed above could arise from incision.

Lastly, we observed that expression of CGRP and P2X3, markers of peptidergic and non-peptidergic sensory neurons, respectively, mainly occurred in small-diameter DRG neurons (<1000 μm^2^; Fig. 4*C*,*D*) in a similar manner to what has been observed in rodent and human DRG (Shiers et al., 2020).

This study highlights that sheep have hitherto untapped potential in mechanistic joint pain research. For example, preclinical orthopedic analysis of potential therapeutics, which tends to focus primarily on histologic assessment of cartilage integrity, could also directly analyze treatment effect on peripheral sensitization by recording the DRG neurons. Additionally, sheep DRG neurons could perhaps serve as better models than those of rodents for testing novel pain therapeutics, since rodents have different metabolic processes of drug breakdown and smaller sized neurons compared with humans (Chakrabarti et al., 2020a). However, to make use of large animals such as sheep fully feasible for pain research, efforts need to be made to develop effective tools. For example, because of the unavailability of specific antibodies that work on sheep tissue, we were unable to assess using IHC if enhanced TRPV1 expression occurred in DRG neurons, as well as being unable to investigate whether P2X3 and CGRP are coexpressed a subset of DRG neurons as has been observed in human DRG (Shiers et al., 2020). Nevertheless, our study paves the way for future investigation of pain mechanisms in OD which might help prevent progression of joint trauma to arthritis.

## Synthesis

Reviewing Editor: Karen Davis, Krembil Research Institute and University of Toronto Decisions are customarily a result of the Reviewing Editor and the peer reviewers coming together and discussing their recommendations until a consensus is reached. When revisions are invited, a fact-based synthesis statement explaining their decision and outlining what is needed to prepare a revision will be listed below. The following reviewer(s) agreed to reveal their identity: NONE.

Basic research into osteroarthritis and joint pain is a growing field with an ever-increasing clinical requirement. The vast majority of this research is performed on rodent models.

This paper shows that a large animal model where the joint more closely resembles human anatomy, can be used to understand mechanisms of nociceptor sensitization. This has high relevance for generation of therapeutics for osteoarthritis pain.

Here the submitted manuscript presents a model of osteochondral defect in sheep. It is imperative that pain models are shown to be effective in non-rodent species and as such this manuscript is of benefit to the field.

This is a nice, and straightforward paper using a sheep model of joint injury to work out mechanisms of nociceptor sensitization in a model where the joint injury more closely resembles what happens in humans. The authors find clear evidence of nociceptor sensitization, including upregulation of TRPV1 expression and function. The authors are to be commended for developing this model in sheep. The field of pain is dominated preclinically by rodent models and there is a growing need for verification of those models in non-rodent species. As such this manuscript is of benefit to the community.

Comments:

1) The time range for the DRG culturing is fairly wide, 2 - 6 weeks. Can the authors show in their plots which recordings came from which time points? I know a formal analysis cannot be done on this sample size, but it would be informative for future work to know if there was a trend to greater changes as time progresses.

2) The differences in CGRP and P2X3 between rodents and humans was not really from the Ray et al paper, it is from Shiers et al in Pain and Journal of Comparative Neurology.

3) There are some little typos here and there. For instance, in the home office approval sentence the University is not named.

4) The discussion could be enhanced with some forward looking statements on how this model can be used to further probe mechanism and why this is such a big improvement over mouse or rat models.

5) Is there any indication that the model caused and pain or discomfort in the experimental animals (other than the lameness measure for the first post-surgical week)? The grimace scale has been successfully adapted to sheep (Hager et al 2017) for example. It would be useful to know whether the peripheral sensitizing effects of the injury are also painful and if so for how long.

6) In the experimental methods the DRGs used for electrophysiology are simply described as ‘lumbar’. Is it know which DRG(s) neurons project to the joint? Using unaffected DRGs would dilute any effect of the injury. In the immunohistochemistry section it states that L3-4 DRGs were used, which are presumably appropriate for the damaged knee?

This would also have been useful for considering the duration of any discomfort as it strikes me as odd that such a large range as 2-6 weeks post-surgery could be used in so few experimental animals. The n is very small for this series of experiments, and its easy to understand why, but 2-6 weeks post-surgery may include very many changes in DRG neuronal receptor expression etc. It would have been far more desirable to have a tighter time course, or at the very least to include a measure that suggest the changes at 2 weeks are the same as those at 6 weeks.

7) The results are promising and show similar responses to those shown in rodent and human DRGs. However it does strike me as somewhat of a missed opportunity not to have been able to record duration of post-operative pain (perhaps the authors have this information from their lameness scoring) and to be more consistent with tissue collection at a set time point. As it is while the data presented is interesting it is difficult to interpret fully and draw and firm conclusions. It is, however, very encouraging that some of the markers and cell populations that are well described in rodents and becoming increasingly so in humans are also present in sheep. This is not sufficient to preclude more rigorous methodology.

8) I don’t consider SEM to be appropriate. Please use SD instead
