# Supplementary figures and images for: Functional Characterization of Ovine Dorsal Root Ganglion Neurons Reveal Peripheral Sensitization after Osteochondral Defect
Source: eNeuro. 2021 Oct 5;8(5):ENEURO.0237-21.2021. doi: 10.1523/ENEURO.0237-21.2021 (PMC8577045; doi:10.1523/ENEURO.0237-21.2021)

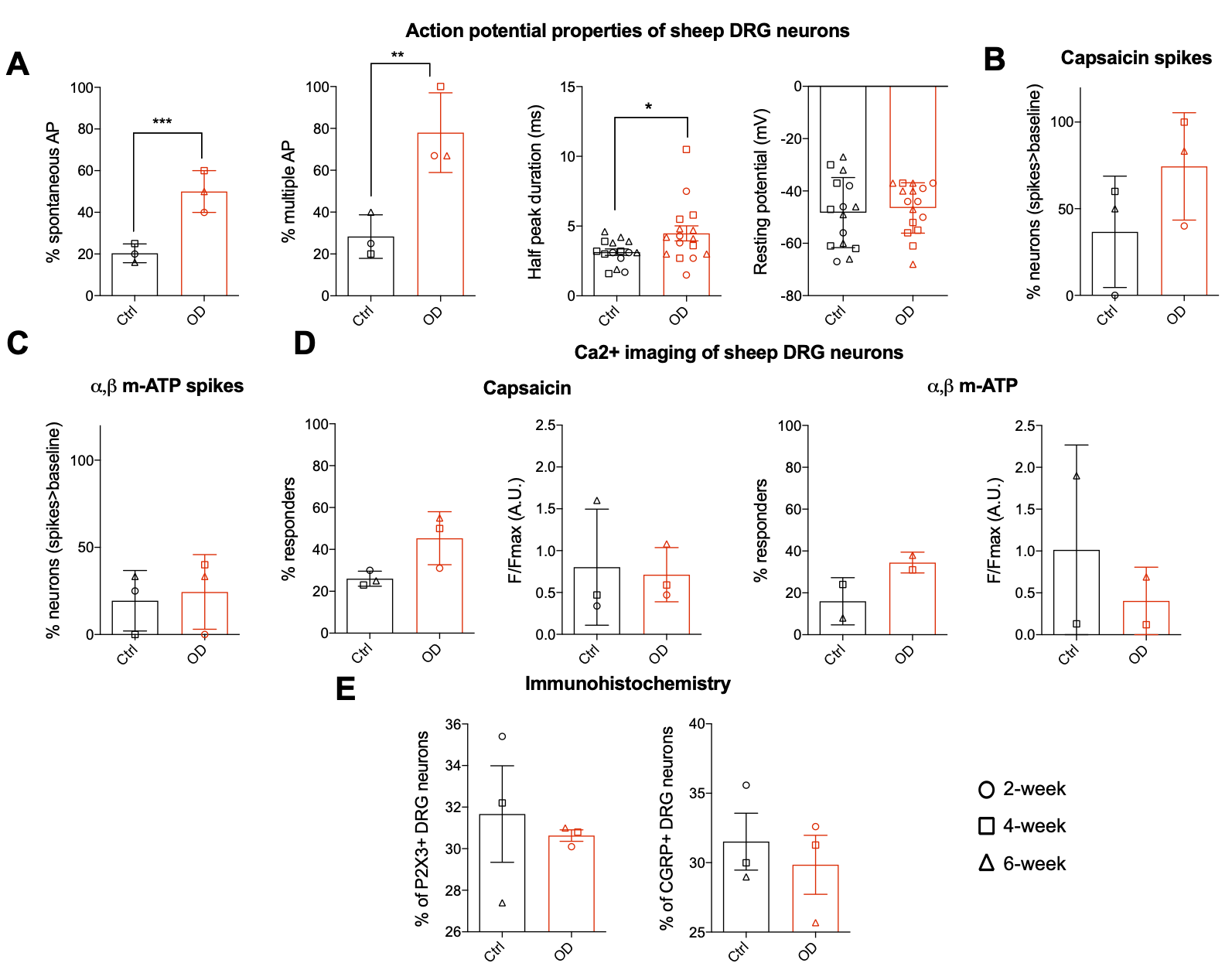

Supplement: Extended Data Figure 2-1 — Extended data supporting Figure 2. Data points from main figures separated according to weeks post-OD. A, AP properties of sheep DRG neurons. Percentage of sheep DRG neurons which evoked AP upon application of capsaicin (B) and αβ m-ATP (C). D, Percentage and magnitude of Ca2+ influx of sheep DRG neurons in response to capsaicin and αβ m-ATP. E, percentage of P2X3 and CGRP positive sheep DRG neurons as assessed using immunohistochemistry. In all the panels, black = control, red = OD, circle = data from sheep two weeks post-OD, square = data from sheep four weeks post-OD and, triangle = data from sheep six weeks post-OD; *p < 0.05, **p < 0.01, unpaired t test. Download Figure 2-1, TIF file. [file enu-eN-NWR-0237-21-s01.tif]
